# Covering Convex Bodies and the Closest Vector Problem

**DOI:** 10.1007/s00454-022-00392-x

**Published:** 2022-05-01

**Authors:** Márton Naszódi, Moritz Venzin

**Affiliations:** 1 MTA-ELTE Lendület Combinatorial Geometry Research Group; Department of Geometry, Loránd Eötvös University, Budapest, Hungary; 2 Institute for Mathematics, École Polytechnique Fédérale de Lausanne, Lausanne, Switzerland

**Keywords:** Closest vector problem, Modulus of smoothness, Lattice sparsification, Convex body in *d*-dimensional space, Approximation, 90C10, 52C07, 68W25, 68Q25, 68U05

## Abstract

We present algorithms for the $(1+\epsilon )$-approximate version of the closest vector problem for certain norms. The currently fastest algorithm (Dadush and Kun 2016) for general norms in dimension *n* has running time of $2^{O(n)}(1/\epsilon )^n$. We improve this substantially in the following two cases. First, for $\ell _p$-norms with $p>2$ (resp. $p \in [1,2]$) fixed, we present an algorithm with a running time of $2^{O(n)}(1+1/\epsilon )^{n/2}$ (resp. $2^{O(n)} (1+1/\epsilon )^{n/p}$). This result is based on a geometric covering problem, that was introduced in the context of CVP by Eisenbrand et al.: How many convex bodies are needed to cover the ball of the norm such that, if scaled by factor 2 around their centroids, each one is contained in the $(1+\epsilon )$-scaled homothet of the norm ball? We provide upper bounds for this $(2,\epsilon )$*-covering number* by exploiting the *modulus of smoothness* of the $\ell _p$-balls. Applying a covering scheme, we can boost any 2-approximation algorithm for CVP to a $(1+\epsilon )$-approximation algorithm with the improved run time, either using a straightforward sampling routine or using the deterministic algorithm of Dadush for the construction of an epsilon net. Second, we consider polyhedral and zonotopal norms. For centrally symmetric polytopes (resp. zonotopes) in ${\mathbb R}^n$ with *O*(*n*) facets (resp. generated by *O*(*n*) line segments), we provide a deterministic $O(\log _2(2+1/\epsilon ))^{O(n)}$ time algorithm. This generalizes the result of Eisenbrand et al. which applies to the $\ell _\infty$-norm. Finally, we establish a connection between the *modulus of smoothness* and *lattice sparsification*. As a consequence, using the enumeration and sparsification tools developped by Dadush, Kun, Peikert, and Vempala, we present a simple alternative to the boosting procedure with the same time and space requirement for $\ell _p$ norms. This connection might be of independent interest.

## Introduction

The *closest vector problem* (CVP) is an important algorithmic problem in the geometry of numbers. Given a rational lattice $\Lambda (A) = \{Ax :x \in \mathbb {Z}^n\}$, with $A \in \mathbb {Q}^{n \times n}$ and a target vector $t \in \mathbb {Q}^n$, the task is to find a close vector in $\mathcal {L}$ to *t* with respect to a given norm. Specifically, given some norm $\Vert \,{\cdot }\,\Vert _K$, a $(1+\epsilon )$-approximation to the closest vector problem, $(1+\epsilon )$-$\text {CVP}_{K}$, is to find a lattice vector whose distance to the target vector is at most $1+\epsilon$ times the minimal distance of the target to the lattice. Whenever *K* is the unit ball of the space $\ell _p^n$ for some $1\le p\le \infty$, we denote the problem by $(1+\epsilon )$-$\text {CVP}_{p}$. The closely related *shortest vector problem* (SVP) asks for the shortest non-zero lattice vector in a given lattice. It was shown that CVP is NP-hard for any $\ell _p$ norm [18] and even NP-hard to approximate up to almost polynomial factors, [7, 15].

The first algorithm to solve integer programming and, in particular, exact $\text {CVP}_{\infty }$ was given by Lenstra [22] with a running time of $2^{O(n^2)}$. His algorithm connects the two fields of geometry of numbers and integer programming. Kannan [21] presented an algorithm for exact $\text {CVP}_{}$ (and $\text {SVP}_{}$) with a running time of $n^{O(n)}$ and polynomial space. Subsequent works improve on the constant in the exponent but improving the running time of $n^{O(n)}$ to single exponential in *n* remained an open problem. After Kannan’s result, it took almost 15 years until Ajtai, Kumar, and Sivakumar presented a randomized algorithm for $\text {SVP}_2$ with time and space $2^{O(n)}$ and $(1+\epsilon )$-$\text {CVP}_{2}$ with time and space $2^{(1+1/\epsilon )n}$ [5, 6]. Subsequently, Blömer and Naewe [9] extended the randomized sieving algorithm of Ajtai et al. to solve $(1+\epsilon )$-$\text {CVP}_{p}$ for all *p* in time $O(1+1/\epsilon )^{2n}$ and space $O(1+1/\epsilon )^n$, see also [3, 27]. For $p = \infty$, Eisenbrand et al. [17] then boosted the algorithm of Blömer and Naewe by showing that $2^{O(n)}\log (2+1/\epsilon )^n$ calls to a $2$-$\text {CVP}_{\infty }$ solver suffice to solve $(1+\epsilon )$-$\text {CVP}_{\infty }$ implying a running time of $O(\log (2+1/\epsilon ))^n$ and space requirement $2^{O(n)}$. Dadush [11] extended the Ajtai–Kumar–Sivakumar sieve to solve $(1+\epsilon )$-$\text {CVP}_{}$ in any norm with a running time of $O(1+1/\epsilon )^{2n}$ and space $O(1+1/\epsilon )^n$. The first single exponential deterministic and exact solver for $\text {CVP}_{2}$ was presented by Micciancio and Voulgaris [25]. Their algorithm needs to store the up to $2(2^n-1)$ facets of the Voronoi cell of the lattice. Recently in [20], Hunkenschröder, Reuland, and Schymura show that this can be avoided and do a first step towards a polynomial space algorithm for $\text {CVP}_{2}$. The currently fastest algorithms for exact $\text {CVP}_{2}$ and $\text {SVP}_2$ use discrete Gaussian sampling and need time and space $2^{n+o(n)}$, see [2, 4]. Despite this progress for the $\ell _2$ norm, for general norms, only the randomized sieving approach seemed available to solve CVP. Using the elegant idea of lattice sparsification, Dadush and Kun [13] presented a deterministic algorithm solving $(1+\epsilon )$-$\text {CVP}_{}$ for any norm in time $2^{O(n)}(1+1/\epsilon )^n$ and with space requirement $2^n {{\,\mathrm{poly}\,}}(n)$—reducing the dependence on $1/\epsilon$ in the running time and removing the dependence on $1/\epsilon$ in the space requirement altogether compared with earlier randomized sieving approaches.

### Our Contribution

In order to devise more efficient algorithms for $\text {CVP}_{K}$ (and, in particular $\text {CVP}_{p}$), we study the problem of how many arbitrarily chosen convex bodies are needed to cover some given convex body *K*, such that when scaled around their respective centroids by a factor 2, each one is contained in $(1+\epsilon )K$. We refer to such a covering as a $(2,\epsilon )$-covering *for* *K*, and the smallest size of such a covering as the $(2,\epsilon )$-covering *number of* *K*.

A key quantity, well studied in the theory of Banach spaces, is the *modulus of smoothness* of a convex body *K*, which expresses how well the boundary of *K* is approximated locally by support hyperplanes, see Definition 3.1.

In this paper the *big oh notation*, $O(\,{\cdot }\,)$, stands for a universal multiplicative constant independent of every other quantity. In particular, we make the dependence on $\epsilon$ and *n* explicit.

- By a standard argument, we show that for any centrally symmetric convex body, a $(2,\epsilon )$-covering is always possible using $2^{O(n)}(1+{1}/{\epsilon })^n$ convex bodies. Then, in Theorem 2.7, we establish a *lower bound* of $2^{-O(n)}(1+{1}/{\epsilon })^{n/2}$ for the Euclidean unit ball.
- For centrally symmetric polytopes (resp. zonotopes) with *m* *facets* (resp. *m* generating line segments), we provide an explicit $(2,\epsilon )$-covering using at most $O(\log (2+{1}/{\epsilon }))^m$ convex bodies, see Propositions 2.5 and 2.6. These are relatively straightforward generalizations of the method of [17] where the cube is considered.
- Our first main result is Theorem 3.2, where it is shown that a bound on the *modulus of smoothness* of *K* yields a *bound on its* $(2,\epsilon )$-covering number. More specifically, if *K* has modulus of smoothness bounded above by $C\tau ^q$, then we find a $(2,\epsilon )$-covering of *K* using $C^{O(n)}(1+1/\epsilon )^{n/q}$ convex bodies. In particular, we obtain a $(2,\epsilon )$-covering for $\ell _p$ balls using $2^{O(n)}(1+1/\epsilon )^{n/2}$ for $p\ge 2$ and $2^{O(n)}(1+1/\epsilon )^{n/p}$ for $p\in [1,2]$, matching the lower bound (Theorem 2.7) for the Euclidean unit ball.
- Our second main result is Theorem 4.2, which shows how a good algorithmic bound on the $(2,\epsilon )$-covering number yields an *efficient* $(1+\epsilon )$-$\text {CVP}_{}$ algorithm. In particular, for norms induced by centrally symmetric polytopes (resp. zonotopes) with *m* facets (resp. generating line segments), the above explicit $(2,\epsilon )$-covering boosts any $2$-$\text {CVP}_{}$ solver for general norms to yield a deterministic $(1+\epsilon )$-$\text {CVP}_{}$ algorithm. This yields an algorithm with running time $O(\log (2+{1}/{\epsilon }))^m$ and $2^n {{\,\mathrm{poly}\,}}(n)$ space, see Corollary 4.3.
- For a centrally symmetric convex body *K* with a certain modulus of smoothness, to avoid the space requirement to depend on the number of convex bodies in the $(2,\epsilon )$-covering of *K*, we show how to *generate a local* $(2,\epsilon )$-covering on the fly. This yields a simple, randomized $(1+\epsilon )$-$\text {CVP}_{p}$ algorithm for $1\le p\le \infty$ with a running time of $O(1+1/\epsilon )^{n/2}$ for $p\ge 2$, and $2^{O(n)}(1+{1}/{\epsilon })^{n/p}$ for $p\in [1,2]$, using $2^{n} {{\,\mathrm{poly}\,}}(n)$ space. Alternatively, we may use an algorithm of Dadush [12] to explicitly enumerate the covering using polynomial space only, derandomizing the algorithm. This is our third main result, see Theorem 4.6. Compared to earlier results in the literature, for instance [9, 13], we improve on the previous best running times of $O(1+1/\epsilon )^n$ for $\ell _p$ norms. Furthermore, our approach immediately generalizes to non-symmetric norms and we obtain a simple CVP solver for $\gamma$-symmetric norms with running time $(1+{1}/({\gamma \epsilon }))^n$ and space requirement $2^{O(n)}$ based on the Ajtai–Kumar–Sivakumar sieve, see Remark 4.7. This almost matches the performance of Dadush and Kun’s algorithm.
- Finally, we establish a connection between *lattice sparsification* and the *modulus of smoothness*, see Lemma 5.2. While the boosting approach described in Sects. 3 and 4 is conceptually very simple and general, and it does not require any knowledge about the approximate $\text {CVP}_{}$  solver used, the proofs are quite technical. We will show that we can tweak the algorithm described by Dadush and Kun in [13] using a simple observation based on the modulus of smoothness in order to obtain the same improved running time for $\text {CVP}_{}$  for norms with a certain modulus of smoothness, in particular $\text {CVP}_{p}$. With this new approach, we restrict ourselves to using lattice sparsification and enumeration and we lose the possibility to use an arbitrary constant approximation $\text {CVP}_{}$-solver. Considering the low space dependency of lattice sparsification and enumeration among all known (single exponential) approximate $\text {CVP}_{}$  solvers and the simplicity of our approach, this might not be a big loss.

It should be noted here that a seemingly similar (with respect to $\epsilon$) bound on the $(2,\epsilon )$-covering number follows from recent work of Arya et al. [8] (see also [1]). Using Macbeath regions, they approximate *any* convex body with a polytope with at most $n^{O(n)}\epsilon ^{-(n-1)/2}$ faces of all dimensions in total, provided that $\epsilon \ll n^{-n}$. It is then straightforward to show that this can be turned into a $(2,\epsilon )$-covering using roughly $n^{O(n)}\epsilon ^{-(n-1)/2}$ convex bodies. Unfortunately, for the purpose of designing approximation algorithms for lattice problems, this is of little use, as already the $n^{O(n)}$ factor is prohibitively high considering that the exact solver of Kannan runs in $n^{O(n)}$ time. Moreover, any approximation based on Macbeath regions requires $\epsilon \ll n^{-n}$, which is too strong a restriction for integer programming related applications. Nonetheless, their result shows that for $\epsilon$ sufficiently small, any convex body admits a $(2,\epsilon )$-covering using $O(1+1/\epsilon )^{n/2}$ convex bodies and raises the question whether the restriction on $\epsilon$ can be removed in general. As mentioned above, in the present work, the dimension *n* is not considered constant, and dependence on it is made explicit everywhere.

The structure of the paper is the following. In Sect. 2, we list basic facts about $(2,\epsilon )$-coverings and prove upper bounds on the $(2,\epsilon )$-covering number of symmetric polytopes and zonotopes (Propositions 2.5 and 2.6). In Theorem 2.7, a lower bound on the covering number of the Euclidean ball is presented. In Sect. 3, it is shown how a bound on the modulus of smoothness yields a bound on the $(2,\epsilon )$-covering number. In Sect. 4, we apply our covering bounds to obtain efficient algorithms for $(1+\epsilon )$-$\text {CVP}_{}$. Finally, Sect. 5 contains Theorem 5.5, which presents another $(1+\epsilon )$-$\text {CVP}_{}$ solver for bodies with a well bounded modulus of convexity, based on efficient lattice sparsification and lattice enumeration algorithms.

The scalar product of two vectors $x=(x_1,\ldots ,x_n)$ and $y=(y_1,\ldots ,y_n)$ in $\mathbb {R}^n$ is denoted by $\langle x,y\rangle =x_1y_1+\dots +x_ny_n$. For a positive integer *k*, we use the notation $[k]=\{1,\ldots ,k\}$.

## $(2,\epsilon )$-Coverings

We denote the *homothetic copy* of a convex body *Q* by factor $\lambda \in \mathbb {R}$ with respect to its *centroid* (also called, center of mass) *c*(*Q*) by $\lambda \odot Q=\lambda (Q-c(Q))+c(Q)$. The following notion is central to our study.

### Definition 2.1

($(2,\epsilon )$-*covering*) For a convex body $K \subseteq \mathbb {R}^n$, a sequence of convex bodies $\{Q_i\}_{i=1}^N$ is a $(2,\epsilon )$-covering if

$$\begin{aligned} K \subseteq \bigcup _{i=1}^N Q_i \subseteq \bigcup _{i=1}^N 2\odot Q_i\subseteq (1+\epsilon )K. \end{aligned}$$

We note that we have fixed the factor 2 for concreteness, we could replace 2 by any other constant. For this reason we will assume $\epsilon \in (0,1)$.

The following three lemmas follow directly from standard packing arguments, we include a proof in the appendix.

### Lemma 2.2

Any origin symmetric convex body $K \subseteq \mathbb {R}^n$ admits a $(2,\epsilon )$-covering by at most $({5}/{\epsilon })^n$ homothetic copies of *K*.

We also note that it is sufficient to consider coverings by centrally symmetric convex bodies only.

### Lemma 2.3

Let *K* be a convex body in $\mathbb {R}^n$ that admits a $(2,\epsilon )$-covering consisting of *N* convex bodies. Then, *K* admits a $(2,\epsilon )$-covering consisting of $10^n N$ centrally symmetric convex bodies.

### Lemma 2.4

Any convex body $K \subseteq \mathbb {R}^n$ with 0 as its centroid has a $(2,\epsilon )$-covering by at most $N =({10}/{\epsilon })^n$ translated copies of $({\epsilon }/{2})(K\cap -K)$.

In the particular case of the cube, in [17], Eisenbrand et al. found a $(2,\epsilon )$-covering that requires $(1+2\log _2(1+1/\epsilon ))^n$ parallelepipeds. The following two propositions show that their method generally works for any zonotope or any centrally symmetric polytope.

A *zonotope* is the Minkowski sum of finitely many line segments, $\mathcal {Z} = \bigl \{ \sum _{i = 1}^m \lambda _i b_i:\lambda _i \in [-1,1], \, \, 1 \le i \le m\bigr \} = \sum _{i=1}^m [-b_i, b_i]$. We refer to the $b_i$ as the *generators* of $\mathcal {Z}$. If $m=n$ and $b_i= e_i$, $i=1,\ldots ,n$, then this zonotope is the unit cube. A zonotope with *m* generators can have up to $$2\left( {\begin{array}{c}m\\ n-1\end{array}}\right) $$ facets; when no *n* of the generators are linearly dependent, this bound is attained, as is not difficult to see.

In the following two propositions, we give upper bounds for the $(2,\epsilon )$-covering of zonotopes with a bounded number of generators and for polytopes with a bounded number of facets. We include these proof in the appendix.

### Proposition 2.5

($(2,\epsilon )$-covering of a zonotope by smaller zonotopes) Let $\mathcal {Z} = \bigl \{\sum _{i=1}^m \lambda _i b_i:\lambda _i \in [-1,1],\,i \in [m]\bigr \}$ be a zonotope with *m* generators, $b_1,\dots ,b_m\in \mathbb {R}^n$. For any $\epsilon >0$, there exists a $(2,\epsilon )$-covering of $\mathcal {Z}$ using $(1+2\log _2(1+1/\epsilon ))^m$ zonotopes.

### Proposition 2.6

($(2,\epsilon )$-covering centrally symmetric polytopes with few facets) Let $P = \{x\in \mathbb {R}^n : |a_i^T x|\le b_i ,\,i \in [m]\}$ be an origin symmetric polytope. There is a $(2,\epsilon )$-covering of *P* using at most $2^m(\log _{4/3}(1/\epsilon )+1)^m$ centrally symmetric convex bodies.

Finally, we prove a lower bound on the $(2,\epsilon )$-covering number of the Euclidean unit ball $B_2^n$ which, by Corollary 3.4, is sharp, up to a logarithmic factor.

### Theorem 2.7

For any $\epsilon \in (0,1/2)$, any $(2,\epsilon )$-covering of the Euclidean unit ball $B_2^n$ consists of at least $2^{-O(n)}(1/\epsilon )^{(n-1)/2}$ convex bodies.

### Proof

Let $\{Q_i\}_{i=1}^N$ be a $(2,\epsilon )$-covering of $B_2^n$ with respective centroids $c_i$. Let $p \in \mathbb {S}^{n-1}$ and let *c* be the centroid of a $Q_i$ such that $p \in Q_i$. First, we show that $\langle p, c \rangle \ge 1-\epsilon$, that is, $Q_i$ is contained in a small solid cap. Suppose by contradiction that $\langle p, c\rangle <1-\epsilon$. By the definition of a $(2,\epsilon )$-covering we need that $\Vert p +(p-c)\Vert \le 1+\epsilon$. This implies $\langle p, p + (p-c)\rangle \le 1+\epsilon$ and we obtain the following contradiction:

$$\begin{aligned} \langle p, p+(p-c)\rangle = 2\langle p, p\rangle + \langle p, -c\rangle > 2 +\epsilon -1 = 1 + \epsilon . \end{aligned}$$

Also by the definition of a $(2, \epsilon )$-covering, we need $\Vert c\Vert \le 1+\epsilon$. Thus, we can show $\Vert p-c\Vert$ is small:

$$\begin{aligned} \langle p-c, p-c\rangle =\langle p, p\rangle + \langle c, c \rangle +2\langle p, -c\rangle \le 1 + (1+\epsilon )^2 + 2(\epsilon -1)\le 5\epsilon . \end{aligned}$$

Thus, for every $Q_i$, $Q_i \cap \mathbb {S}^{n-1}$ is contained in a cap of radius $\sqrt{5\epsilon }$. Denoting by $\sigma (\,{\cdot }\,)$ the uniform probability measure on the sphere, this means that for any convex body $Q_i$ in the $(2,\epsilon )$-covering , $\sigma (Q_i) \le 2^{O(n)}\epsilon ^{(n-1)/2}$ (cf. [10, Lem. 3.1]). Since a $(2,\epsilon )$-covering of $B_2^n$ needs to cover all of $\mathbb {S}^{n-1}$, we obtain the desired lower bound on *N*. $\square$

## $(2,\epsilon )$-Coverings via Modulus of Smoothness

For a convex body *K*, we will consider its *gauge function* $\left\| \,{\cdot }\,\right\| _K$, defined by $\left\| x\right\| _K = \inf {\{s :x \in sK\}}$. If *K* is origin symmetric, then $\left\| \,{\cdot }\,\right\| _K$ defines a norm.

### Definition 3.1

(*modulus of smoothness*) The *modulus of smoothness* of an origin-symmetric convex body *K*, $\rho _K(\tau ):(0,1) \rightarrow (0,1)$, is defined by

$$\begin{aligned} \rho _K(\tau ) = \frac{1}{2} \sup _{\left\| x\right\| _K = \left\| y\right\| _K = 1}(\left\| x+\tau y\right\| _K + \left\| x-\tau y\right\| _K-2). \end{aligned}$$

We remark first that any origin symmetric body *K* has modulus of smoothness $\rho _K(\tau ) \le \tau$, this follows from the subadditivity of the norm. The modulus of smoothness of *K* measures how well *K* can be locally approximated by hyperplanes: If $\Vert x\Vert _K = 1$ and $\Vert \tau y\Vert _K = \tau$ and both $x+y$ and $x-y$ lie on a support hyperplane of *K* at *x*, then both $\Vert x+\tau y\Vert _K,\Vert x-\tau y\Vert _K \ge 1$, but we also have the upper bound of

$$\begin{aligned} \Vert x\pm \tau y\Vert _K \le 1 + 2\rho _K(\tau ). \end{aligned}$$

If $\rho _K(\tau )$ can be bounded by a polynomial of degree higher than 1, say $\tau ^2$, then $x\pm \tau y$ are closer to the boundary of *K* compared to what subadditivity, $\Vert x\pm \tau y\Vert _K \le \Vert x\Vert _K + \Vert \tau y\Vert _K$, alone yields. Still assuming $\rho _K(\tau ) \le \tau ^2$ and letting $\epsilon \in (0,1)$, this means that all points $y\in K$ with $\Vert x-y\Vert \le \sqrt{\epsilon }$ are approximated up to an additive $\epsilon$ by the tangential hyperplane at *x*. This behaviour of some norms is exploited in the next theorem.

### Theorem 3.2

Let $K \subseteq \mathbb {R}^n$ be an origin symmetric convex body, and $\epsilon \in (0,1)$. Assume that the modulus of smoothness of *K* is bounded by

$$\begin{aligned} \rho _K(\tau ) \le C\tau ^q \end{aligned}$$

with some constants $C,q>1$. Then, there exists a $(2,\epsilon )$-covering of *K* consisting of

$$\begin{aligned} 2^{O(n)}\log \biggl (1+\frac{1}{\epsilon }\biggr )\biggl (\frac{C}{\epsilon }\biggr )^{\!n/q}+O(C)^{n/(q-1)} \end{aligned}$$

centrally symmetric convex bodies. The encoding length of each such body is a polynomial in the encoding length of *K*.

**Fig. 1:**
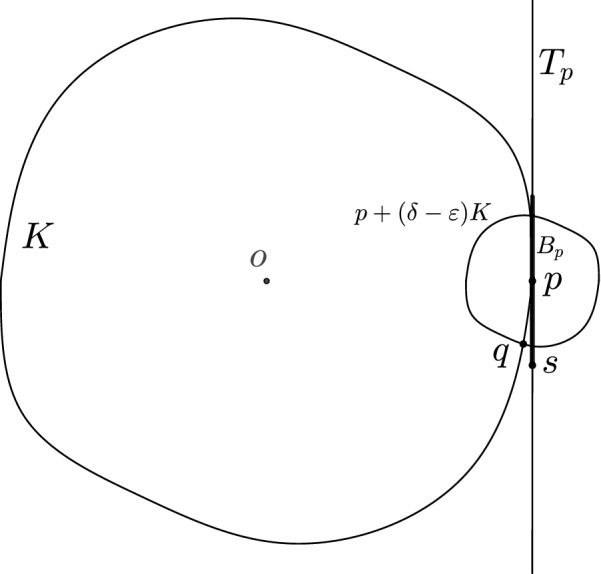
Proof of (1)

### Proof

Set $\delta =({\epsilon }/{C})^{1/q}/4$. We may assume that $\epsilon \le ({1}/({8C^{1/q}}))^{q/(q-1)}$, in which case $\delta -\epsilon \ge \delta /2$. Otherwise, we may apply Lemma 2.2 and obtain a $(2,\epsilon )$-covering of *K* consisting of $O(C)^{n/(q-1)}$ bodies. We denote $\left\| \,{\cdot }\,\right\| _K$ by $\left\| \,{\cdot }\,\right\|$.

We first describe a $(2,2\epsilon )$-covering of *K* only in the neighborhood of a point and then, using a packing argument, we extend this construction to obtain a $(2,2\epsilon )$-covering for all of *K*.

Fix a point *p* on the boundary of *K* that is, $\left\| p\right\| = 1$. Denote by $T_p$ a supporting hyperplane of *K* at *p*. Let $B_p$ be the intersection of $T_p$ with $p + \delta K$, i.e., $B_p := T_p \cap \{x : \left\| x-p\right\| \le \delta \}$.

First, we show that

1 $$\begin{aligned} \mathrm{bd}\mathrm{({K})} \cap (p+(\delta - \epsilon )K)\subseteq {{\,\mathrm{conv}\,}}(0, B_p). \end{aligned}$$

Indeed, let *q* be a point in $\mathrm{bd}\mathrm{({K})} \cap (p+(\delta -\epsilon )K)$, and let *L* denote the two-dimensional linear plane spanned by *p*, *q* and the origin *o*, see Fig. 1. Clearly, $L\cap T_p$ is a line, and there are two points on this line at distance $\delta$ from *p*. Let *s* denote the point of these two which is on the same side of the line *op* as *q*. That is, *s* is a point on the lateral surface of the cone ${{\,\mathrm{conv}\,}}(0, B_p)$. By the assumption on the modulus of smoothness of *K*, we have $s^\prime :=s/\left\| s\right\|$ is at distance at most $\epsilon$ from *s* (a detailed computation of a similar fact is given below in this proof). Thus,

2 $$\begin{aligned} \left\| s^\prime -p\right\| \ge \delta -\epsilon . \end{aligned}$$

Now, *L* is a normed plane with unit circle $K\cap L$ and *p* is a unit vector in *L*. It is a classical fact in the theory of normed planes [24, Prop. 31] that as a point moves along the curve $K\cap L$ starting at *p* and ending at $-p$, the distance (w.r.t. $\Vert \,{\cdot }\,\Vert _K$) of the moving point to *p* is increasing. Thus, by (2), the arc of $K\cap L$ between *p* and $s^\prime$ contains *q*, which yields that *q* is in the cone ${{\,\mathrm{conv}\,}}(0, B_p)$, proving (1).

Next, instead of the cone ${{\,\mathrm{conv}\,}}(0, B_p)$, we will consider the cylinder

$$\begin{aligned} C_p = B_p + [0, -p]. \end{aligned}$$

Clearly, we have ${{\,\mathrm{conv}\,}}(0, B_p) \subseteq C_p$. We may assume that $\epsilon$ is of the form $\epsilon =(2^{k}-1)^{-1}$, where *k* is a positive integer. For $i \in [k]$, consider the following slice of $C_p$:

3 $$\begin{aligned} C_p(i) = \bigl (B_p +[-(2^i-1)\epsilon p, -(2^{i-1}-1)\epsilon p]\bigr ). \end{aligned}$$

Clearly, $2\odot C_p(i)\subseteq \widehat{C_p} := 2\odot B_p + [\epsilon p,-{3p}/{2}]$ and the centroid $c(C_p(i))$ is at $(1-({3}\cdot 2^{i-1}/{2}-1))\epsilon p$ for each $i\in [k]$. We claim that $\widehat{C_p}\subseteq (1+2\epsilon )K$. Since $\delta \le 1/4$ and $K=-K$, we have $2\odot B_p -{3p}/{2}\subseteq K$. Thus, it suffices to check that $2\odot B_p+\epsilon p\subseteq (1+2\epsilon )K$.

Let $x\in 2\odot B_p + \epsilon p$, i.e., $x = p + 2(z-p) + \epsilon p$ for some $z\in B_p$. We will show that $\Vert p+2(z-p)\Vert \le 1+2\epsilon$. Since both *p* and *z* lie in $T_p$, then so do $p + 2(z-p)$ and $p + 2(p-z)$, and thus, we have $\Vert p+2(z-p)\Vert , \Vert p + 2(p-z)\Vert \ge 1$. One has $\Vert 2(z-p)\Vert \le 2\delta =({\epsilon }/{C})^{1/q}/2$ and so by the assumption on the modulus of smoothness of *K*, we obtain

$$\begin{aligned} \Vert p + 2(z-p)\Vert \le 2C\Vert 2(z-p)\Vert ^q + 1\le 1 + \epsilon . \end{aligned}$$

Thus, $\widehat{C_p} \subseteq (1+2\epsilon )K$, and hence,

$$\begin{aligned} 2\odot C_p(i) \subseteq (1+2\epsilon )K \end{aligned}$$

for each $i \in [k]$. Since, by (1), all points on the boundary of *K* at distance at most $\delta - \epsilon$ from *p* are covered by $C_p$, we see that all points *x*, such that $\Vert {x}/{\Vert x\Vert }-p\Vert \le \delta - \epsilon$ are covered by one of the slices of $C_p$. Thus, in order to extend the above construction to a $(2,2\epsilon )$-covering of *K*, we pick points $\{p_i\}_{i=1}^N$ on the boundary of *K* such that $\mathrm {bd}\left( K\right) \subseteq \bigcup _{i=1}^N p_i + (\delta - \epsilon )K$. By Lemma 2.2,

$$\begin{aligned} N =2^{O(n)}\biggl (\frac{1}{\delta - \epsilon }\biggr )^{\!n} =2^{O(n)}\biggl (\frac{C}{\epsilon }\biggr )^{\!n/q} \end{aligned}$$

such points suffice.

Thus, we obtain a $(2,2\epsilon )$-covering for *K* by constructing $C_{p_i}$ for each $i \in [N]$ and slicing each $C_{p_i}$ as in (3). Finally, replacing $\epsilon$ by ${\epsilon }/{2}$, we indeed get a $(2,\epsilon )$-covering of *K* using $2^{O(n)}({C}/{\epsilon })^{n/q}\log ({1/\epsilon })$ convex bodies, each described by a polynomial in the encoding length of *K*, see [19]. $\square$

### Theorem 3.3

(modulus of smoothness for $\ell _p$ spaces [23]) We have

$$\begin{aligned}\rho _{\ell _p}(\tau ) ={\left\{ \begin{array}{ll} (((1+\tau )^p + (1-\tau )^p)/2)^{1/p}-1 \le 2^p\tau ^2,&{} \text {if } 2 \le p < \infty ,\\ (1+\tau ^p)^{1/p}-1 \le \tau ^p/p, &{}\text {if } 1\le p \le 2. \end{array}\right. } \end{aligned}$$

### Proof

By [23, end of Sect. 2], we only need to show $(((1+\tau )^p + |1-\tau )^p)/2)^{1/p}-1 \le 2^p\tau ^2$ for $\tau \in (0,1)$ and $2\le p<\infty$. By computing

$$\begin{aligned} \frac{\mathrm {d}}{\mathrm {d}p}\bigl [ (1+\tau )^p + (1-\tau )^p\bigr ],\quad \text {and then}\quad \frac{\mathrm {d}}{\mathrm {d}\tau }\frac{\mathrm {d}}{\mathrm {d}p}\bigl [ (1+\tau )^p + (1-\tau )^p \bigr ], \end{aligned}$$

one obtains that $(1+\tau )^p + (1-\tau )^p\le (1+\tau )^{\left\lceil p \right\rceil } + (1-\tau )^{\left\lceil p \right\rceil }$. Next, by taking the binomial expansion, one checks that $\bigl [(1+\tau )^{\left\lceil p \right\rceil } + (1-\tau )^{\left\lceil p \right\rceil }\bigr ]\le (1+2^p\tau ^2)^{\left\lfloor p \right\rfloor }$, completing the proof. $\square$

Theorems 3.2 and 3.3 imply the following.

### Corollary 3.4

($(2,\epsilon )$-coverings for $\ell _p$ balls) For small enough $\epsilon$, there exists a $(2,\epsilon )$-covering for $\ell _p$ balls using $2^{O(n)}\log (1+1/\epsilon )({1}/{\epsilon })^{n/2}$ convex bodies for $2 \le p < \infty$ and $2^{O(n)}\log (1+1/\epsilon )({1}/{\epsilon })^{n/p}$ convex bodies for $1\le p \le 2$.

## Using $(2,\epsilon )$-Coverings for the Closest Vector Problem

We first recall the goal and some important notions of this section: We are given a rational lattice $\Lambda (A) = \{Ax : x \in \mathbb {Z}^n\}$, with $A \in \mathbb {Q}^{n \times n}$ and a target vector $t \in \mathbb {Q}^n$, and we would like to solve $(1+\epsilon )$-approximate $\text {CVP}_{K}$, i.e., find a lattice vector $v \in \Lambda (A)$ such that $\Vert v-t\Vert _K \le (1+\epsilon )\min _{w\in \Lambda (A)}\Vert w-t\Vert _K$. $\Vert \,{\cdot }\,\Vert _K$ is defined by $\Vert x\Vert _K = \inf {\{s :x\in sK\}}$, if *K* is origin symmetric and convex, this defines a norm. If 0 is not the center of symmetry but in the interior of *K* then we lose the symmetry, i.e., $\Vert x\Vert _K \ne \Vert -x\Vert _K$. We denote by *b* the encoding length of the relevant input: $A,t,\epsilon$, encoding length of *K*, etc.

In this section, we will first describe how a $(2,\epsilon )$-covering for *K* using *N* convex bodies boosts any $2$-$\text {CVP}_{}$ solver for general norms to a $(1+\epsilon )$-$\text {CVP}_{K}$ solver at the expense of a factor $N 2^{O(n)}{{\,\mathrm{poly}\,}}(b,{1}/{\epsilon })$ in the running time. This algorithm, together with the construction of Propositions 2.5 and 2.6 directly implies a $(1+\epsilon )$-$\text {CVP}_{}$ solver for polytopes and zonotopes with running time of $2^{O(n+m)}(\log (1+1/\epsilon ))^{m}$ times some polynomial in *b* and *n* and with space requirement that of the $2$-$\text {CVP}_{}$ solver used.

Next, we are going to adapt the construction of Theorem 3.2 to yield a randomized algorithm, that for some fixed point $p\in K$, generates a local $(2,\epsilon )$-covering for *K* containing *p*. This yields a randomized $(1+\epsilon )$-$\text {CVP}_{}$ solver with the improved running time for $\ell _p$ norms and with space requirement only depending on that of the 2-approximate $\text {CVP}_{}$ solver used. This construction can also be derandomized.

The boosting procedure we are going to describe assumes that we are able to sample uniformly within *K* and that we can calculate a separating hyperplane at any point on the boundary of *K*. However, if only a weak membership and a weak separation oracle is provided, the procedure can be adapted such that it suffices to sample almost uniformly, see the algorithm of Dyer et al. [16], and to only calculate a weakly separating hyperplane. We neglect this implementation detail.

As for the convex body *K*, we assume that $n^{-3/2}B_2^n \subseteq K \subseteq B_2^n$, and thus,

4 $$\begin{aligned} \Vert x\Vert _2 \le \Vert x\Vert _K \le n^{3/2}\Vert x\Vert _2. \end{aligned}$$

This can be ensured by applying an affine transformation, which is polynomial in the input size of *K*, to both *K* and the lattice $\Lambda (A)$, see [19].

For concreteness, we choose to use the elegant and currently fastest algorithm for general norms by Dadush and Kun as our $2$-$\text {CVP}_{}$ solver.

### Theorem 4.1

(approximate CVP in any norm [13])  There exists a deterministic algorithm that for any norm $\left\| \,{\cdot }\,\right\| _K$, *n*-dimensional lattice $\Lambda (A)$ and for any target $t\in \mathbb {R}^n$, computes $y \in \Lambda (A)$, a $(1+\epsilon )$-approximate minimizer to $\left\| t-x\right\| _K$, $x \in \Lambda (A)$, in time $O({{\,\mathrm{poly}\,}}(n,b)2^{O(n)}(1+{1}/{\epsilon })^n)$ and $O({{\,\mathrm{poly}\,}}(n,b)2^n)$ space.

### Theorem 4.2

(boosting 2-CVP using a $(2,\epsilon )$-covering)  Assume we are given an origin symmetric convex body *K* in $\mathbb {R}^n$ and a $(2,\epsilon )$-covering for *K* consisting of *N* convex bodies. Then we can solve the $(1+7\epsilon )$-$\text {CVP}_{K}$ for $\Lambda (A)$ and target $t\in \mathbb {Q}^n$ with $O(N\log (1+{1}/{\epsilon })(\log n+ \log b))$ calls to a 2-approximate $\text {CVP}_{}$ solver for general norms.

### Proof

We may multiply $\Lambda (A)$ and *t* by the least common multiple of the denominators of the $n^2$ entries of *A* and the *n* entries of *t*. The resulting lattice and target are integral, $\Lambda (\tilde{A}) \in \mathbb {Z}^{n \times n}$ and $\tilde{t} \in \mathbb {Z}^n$. Since the lowest common multiple is bounded by $2^{(n^2 + n)b}$, the resulting basis of $\tilde{A}$ has Euclidean length at most $2^{(n^2 + n)b}$. Assuming $t\notin \Lambda (A)$, we see that

$$\begin{aligned} 1 \le \min _{x \in \Lambda (\tilde{A})}\Vert x-\tilde{t}\Vert _2 \le n2^{(n^2 +n)b}. \end{aligned}$$

By our assumption (4), we have

$$\begin{aligned} 1 \le \min _{x \in \Lambda (A)}\Vert x-t\Vert _K \le n^{5/2}2^{(n^2 + n)b}. \end{aligned}$$

Let $\{Q_i+c_i\}_{i=1}^N$ be the given $(2,\epsilon )$-covering for *K*, where the origin is the centroid of each of the $Q_i$.

For our algorithm, for any norm $\Vert \,{\cdot }\,\Vert _Q$, we assume that the 2-approximate $\text {CVP}_{Q}$ algorithm that we use with target *t* only returns a lattice vector *v* if $\Vert t-v\Vert _Q \le 2$.

We want to find *f* such that $c_i+(1+\epsilon )^f Q_i$ contains a lattice vector for some $i\in [N]$, but $c_i+(1+\epsilon )^{f-1} Q_i$ contains no lattice vector for any $i\in [N]$. As in [17], we apply a binary search for *f*.

(i) Initialize $$L \leftarrow 0$$, $$U \leftarrow \bigl \lceil \log _{1+\epsilon }n^{5/2} 2^{(n^2 + n)b}\bigr \rceil $$ and $$x = 0$$.
(ii) While $U-L \ge 4$, do a binary search step:

- For all $i \in [N]$, solve a 2-approximate $\text {CVP}_{(1+\epsilon )^{L +\lceil (U-L)/2\rceil }Q_i}$ problem with target $(1+\epsilon )^{L +\lceil (U-L)/2\rceil }c_i + t$.
- If some lattice vector *v* is returned, update $U \leftarrow \lceil \log _{1+\epsilon }\Vert v-t\Vert _K \rceil$ and $x \leftarrow v$.
- Otherwise, update $$L \leftarrow L+\lceil (U-L)/2\rceil $$.

(iii) Return *x*.

It is immediate that for any $\lambda >0$, $\{\lambda Q_i+\lambda c_i\}_{i=1}^N$ is a $(2,\epsilon )$-covering for $\lambda K$. Thus if, for some *L* and *U* at step (iib), no lattice vector *v* is returned, then

$$\begin{aligned} t + (1+\epsilon )^{L + \lceil (U-L)/2\rceil }K \subseteq t + \bigcup _{i=1}^N(1+\epsilon )^{L + \lceil (U-L)/2\rceil }(c_i + Q_i) \end{aligned}$$

contains no lattice vector, and so $\min _{v \in \Lambda (A)}\Vert v-t\Vert _K \ge (1+\epsilon )^{L + \lceil (U-L)/2\rceil }$. In the case a lattice vector is returned, then

$$\begin{aligned} \min _{x \in \Lambda (A)}\Vert t-x\Vert _K \le \Vert v-t\Vert _K \le (1+\epsilon )^{L +\lceil (U-L)/2\rceil + 1} \end{aligned}$$

since the $Q_i$ are a $(2,\epsilon )$-covering of *K*. Since *U* and *L* are valid upper and lower bounds for *f* at the beginning of the algorithm, we see that throughout the algorithm, the following invariant is maintained:

$$\begin{aligned} (1+\epsilon )^L \le \min _{v \in \Lambda (A)}\Vert v-t\Vert _K \le (1+\epsilon )^U. \end{aligned}$$

If the algorithm terminates, then $U-L \le 3$ since *U* and *L* are both integers. Thus, because of the above invariant, the lattice vector $x \in \Lambda (A)$ returned satisfies

$$\begin{aligned} \Vert x-t\Vert _K&\le (1+\epsilon )^U \le (1+\epsilon )^{L+3} \le (1+\epsilon )^3\min _{v \in \Lambda (A)}\Vert v-t\Vert _K \\&\le (1+7\epsilon )\min _{v \in \Lambda (A)}\Vert v-t\Vert _K. \end{aligned}$$

It remains to be shown that the binary search terminates in $O((\log n+\log b)/{\epsilon })$ steps. Indeed, for some *U* and *L*, let $U_{new },L_{new }$ be the *U* and *L* after having executed step (ii) once. If $U-L \ge 6$, it is straightforward to check that $U_{new }-L_{new } \le {3}(U-L)/4$. If $4 \le U-L \le 5$, $U_{new }-L_{new } \le (U-L)-1$. Since $U-L \le \log _{1+\epsilon }(n^{5/2}2^{(n^2 + n)b})$ at the beginning of the algorithm, we are done after $\log _{5/4}(\log _{1+\epsilon }(n^{5/2}2^{(n^2 +n)b})) = O(\log (1+{1}/{\epsilon })(\log n + \log b))$ iterations. $\square$

### Corollary 4.3

($(1+\epsilon )$-approximate $\text {CVP}$ for polytopes and zonotopes) Let *K* be a full-dimensional origin symmetric polytope with *m* facets or a full-dimensional zonotope with *m* generators (in particular, $m\ge n$). Then for any $\epsilon \in (0,1)$, the $(1+\epsilon )$-approximate $\text {CVP}_{K}$ problem can be solved deterministically in time $O({{\,\mathrm{poly}\,}}(n,b,{1}/{\epsilon })2^{O(n+m)}\log (1+1/\epsilon )^{m})$ and space $O({{\,\mathrm{poly}\,}}(n)2^n)$.

### Proof

Replace $\epsilon$ by $\epsilon /7$ and run the algorithm in Theorem 4.2 on a $(2,\epsilon )$-covering of *K* constructed in the proof of Propositions 2.5 or 2.6. To avoid a space requirement depending on the number of convex bodies *N* required in the $(2,\epsilon )$-covering for *K*, every time we call step (iia) of the algorithm, for each $i \in [N]$, we first calculate $Q_i$ and then run the appropriately scaled 2-approximate $\text {CVP}_{}$ instance. $\square$

### Remark 4.4

The preceding corollary is the reason why we opted to describe a $(2,\epsilon )$-covering with symmetric convex bodies for symmetric polytopes in Proposition 2.6: The algorithm of Dadush and Kun can handle non-symmetric norms $\Vert \,{\cdot }\,\Vert _K$, provided 0 is in some sense “close” to the centroid of *K*, for more details see [13]. Since calculating deterministically the centroid is a hard problem and no efficient algorithms are known, see [28], we would most likely have to resort to a randomized algorithm to approximate the centroid which in turn randomizes our boosting procedure.

### Theorem 4.5

(local $(2,\epsilon )$-covering) Let *K* be an origin symmetric convex body such that $\Vert \,{\cdot }\,\Vert _K$ has modulus of smoothness $C\tau ^q$ for $C, q > 1$ and $\epsilon \in (0,1)$. Then, in polynomial time, we can find at most $O(\log (1+1/\epsilon ))$ origin symmetric convex bodies $\{Q_i\}$ and translations $\{c_i\}$ such that for some constant $c > 0$:

- For all *i*, $$c_i + 2Q_i \subseteq (1+\epsilon )K$$.
- For $q \in K$, the probability that *q* is contained in $c_i + Q_i$ for some *i* is greater than $\min {(2^{-cn}C^{-n/q}\epsilon ^{-n/q},({8^qC})^{-n/(q-1)})}$.

### Proof

Set $\epsilon \leftarrow \epsilon /3$. If $\epsilon >({1}/({8C^{1/q}}))^{q/(q-1)}$, we uniformly sample a point *x* from $(1+\epsilon )K$ and return $\epsilon K$ and *x*. Any point in *K* has probability greater or equal than

$$\begin{aligned} \biggl (\frac{\epsilon }{1+\epsilon }\biggr )^{\!n} \end{aligned}$$

of being covered by $x + \epsilon K$.

If $\epsilon \le ({1}/({8C^{1/q}}))^{q/(q-1)}$, similarly as in Theorem 3.2, we set $\delta =({\epsilon }/{C})^{1/q}/4$. We uniformly sample a point *x* from $(1+\delta /4)K$. Let $p ={x}/{\Vert x\Vert }$ and for $i \in [{\log (1/\epsilon )}]$, consider the slices $C_p(i)$ of $C_p$ as in (3) in the proof of Theorem 3.2. For all such $C_p(i)$, denoting by $c(C_p(i))$ its centroid, we return the origin symmetric convex bodies $\{C_p(i)-c(C_p(i))\}$ and the translations $\{c(C_p(i))\}$.

Next, fix a point $q \in K$. With probability greater or equal to

$$\begin{aligned} \frac{1}{2}\cdot \frac{(\delta /4)^n}{(1+\delta /4)^n}\quad \quad \text { we have that }\quad \left\| \frac{q}{\left\| q\right\| } - x\right\| \le \frac{\delta }{4}. \end{aligned}$$

In that case, $\left\| {q}/({\left\| q\right\| }- p)\right\| \le \delta /2 \le \delta -\epsilon$ and thus, $C_p$ as in (3) of Theorem 3.2 contains *q*. It follows that for some $c >0$ independent of *n*, *C*, and *q*, with probability greater or equal to $2^{-cn}C^{-n/q}\epsilon ^{n/q}$ one of the cylinders $C_p(i)$ contain *q*. $\square$

The next theorem combines the algorithms of Theorems 4.5 and 4.2 to yield an efficient $(1+\epsilon )$-approximate CVP solver for norms with a well bounded modulus of smoothness.

### Theorem 4.6

(boosting 2-CVP for a body with small modulus of smoothness) Let *K* be a origin symmetric convex body with modulus of smoothness

$$\begin{aligned} \rho _K(\tau )\le C\tau ^q,\quad \ \ \ \text { with }\quad C,q >1. \end{aligned}$$

Then the algorithm presented in the proof solves $(1+\epsilon )$-$\text {CVP}_{K}$ with probability at least $1-2^{-n}$. Its running time is $O({{\,\mathrm{poly}\,}}(n,b,\log (1/\epsilon ))(2^{O(n)}C^{n/q}(1/\epsilon )^{n/q}+O(C)^{n/{(q-1)}}))$, and the space requirement is equal to that of a $2$-$\text {CVP}_{}$ solver that handles any norm.

### Proof

We set $\epsilon \leftarrow \epsilon /7$ and without loss of generality, we may assume

$$\begin{aligned} 1 \le \min _{x \in \Lambda (A)}\Vert x-t\Vert _K \le n^{5/2}2^{(n^2 + n)b}. \end{aligned}$$

We again assume that, for any norm $\left\| \,{\cdot }\,\right\| _Q$, the $2$-$\text {CVP}_{Q}$ with target *t* only returns a lattice vector *v* if $\Vert t-v\Vert _Q \le 2$, if there is no such *v*, it returns nothing.

We adapt the algorithm of Theorem 4.2:

(i) Initialize $$L \leftarrow 0$$, $$U \leftarrow \lceil {\log _{1+\epsilon }n^{5/2} 2^{(n^2 + n)b}}\rceil $$ and $$x = 0$$.
(ii) While $U-L \ge 4$, do a binary search step:

- Run the algorithm from Theorem 4.5 and denote the returned convex bodies and translations by $Q_i$ and $c_i$ respectively. For all *i*, solve a 2-approximate $\text {CVP}_{(1+\epsilon )^{L +\lceil (U-L)/2\rceil }Q_i}$ problem with target $(1+\epsilon )^{L +\lceil (U-L)/2\rceil }c_i + t$. Repeat *N* times.

(iib) If some lattice vector *v* is returned, update $U \leftarrow \lceil {\log _{1+\epsilon }\Vert v-t\Vert _K }\rceil$ and $x \leftarrow v$.
(iic) Otherwise, update $L \leftarrow L+\lceil (U-L)/2\rceil$.

(iii) Return *x*.

Correctness of the algorithm follows from Theorem 4.2, provided step (ii) runs correctly (i.e., correctly detects whether there is a lattice point or not with high probability) for all $O(\log ({1}/{\epsilon })(\log n+\log b))$ iterations. To verify this, let $v \in \mathcal {L}$ be some lattice vector contained in a homothet of *K* at some fixed iteration of the algorithm. With probability $p =2^{-cn}C^{-n/q}(1/\epsilon )^{n/q}$ or $({1}/({8^q C}))^{1/(q-1)}$ respectively, one of the convex bodies returned by one run of Theorem 4.5 contains *v*. Thus, repeating step (iia) $n(2^{cn}C^{n/q}(1/\epsilon )^{n/q} + (8^qC)^{1/(q-1)})$ times, with probability greater than $1-2^{-n}$, *v* is contained in one of the convex bodies returned and step (ii) runs correctly. Since step (ii) needs to run correctly each of the $O(\log ({1}/{\epsilon })(\log n+\log b))$ iterations necessary to find the correct *U* and *L*, by the union bound, it is sufficient to set $N =O(n\log (\log ({1}/{\epsilon })(\log n+\log b))2^{cn}C^{n/q}(1/\epsilon )^{n/q} + (8^q C)^{1/(q-1)})$ to guarantee a success probability of $1-2^{-n}$. This implies the bound on the running time. $\square$

In our proof of Theorem 4.6, instead of applying our local covering algorithm, Theorem 4.5, we could use a recent result of Dadush [12, Thm. 4.1]. There, a deterministic algorithm is presented to build and iterate over an epsilon net in $2^{O(n)}(1+1/\epsilon )^n$ time and ${{\,\mathrm{poly}\,}}(n)$ space. For symmetric convex bodies with modulus of smoothness bounded by $C\tau ^q$, we may apply this result with $O(\epsilon ^{1/q})$, as in Theorem 4.5, in place of $\epsilon$ to build a covering of size $O({1}/{\epsilon })^{n/q}$. This would replace the sampling part in Theorem 4.5 and thus derandomizes our boosting procedure.

### Remark 4.7

One may consider convex bodies that are not necessarily origin symmetric. Assume that a convex body *K* is $\gamma$ *-symmetric*, that is, ${{\,\mathrm{vol}\,}}(K \cap -K) \ge \gamma ^n{{\,\mathrm{vol}\,}}(K)$. Then the result of Dadush and Kun (Theorem 4.1) still applies (see [13]), and it is straightforward to modify the above algorithm to obtain a $(1+\epsilon )$-approximate CVP algorithm for $\left\| \,{\cdot }\,\right\| _K$ using $2^{O(n)}({1}/({\gamma \epsilon }))^n$ calls to a 2-approximate CVP algorithm handling any symmetric norm, for instance the AKS based algorithm of Dadush [11], resulting in an algorithm with time $O({1}/({\gamma \epsilon }))^n$ and space $2^{O(n)}$. We essentially use Theorem 4.5 with $q = 1$: we sample a point *p* in $(1+\epsilon /3)K$ and output $({\epsilon }/{3})(K \cap -K)$ and *p*. Thus, each point in *K* has probability greater or equal to $2^{-O(n)}({1}/({\gamma \epsilon }))^n$ of being covered.

## Sparsifiers and the Modulus of Smoothness

In this section we describe a surprising connection between lattice sparsifiers as used by Dadush and Kun and the modulus of smoothness. Informally, our main technical contribution is the observation that for a lattice-point-free convex body *K* with modulus of smoothness bounded by $C\tau ^q$, a $O(\epsilon ^{1/q})$-sparsifier for *K* preserves the metric information up to an additive error of $O(\epsilon )$. We will show that we can tweak the algorithm of Dadush and Kun using this simple observation in order to match the running time of the preceding boosting procedure. We will only consider origin symmetric-convex bodies $K\subseteq \mathbb {R}^n$.

### Definition 5.1

(*lattice sparsifier for origin symmetric* *K* [13])  Let $K \subseteq \mathbb {R}^n$ be an origin-symmetric convex body, $\mathcal {L}$ be an *n*-dimensional lattice and $\delta > 0$. A $(K,\delta )$ sparsifier for $\mathcal {L}$ is a sublattice $\mathcal {L'}\subseteq \mathcal {L}$ satisfying

- $$G(K, \mathcal {L'}) \le O({1}/{\delta })^n$$,
- $$\forall \, x \in \mathbb {R}^n$$, $$d_K(\mathcal {L'},x) \le d_K(\mathcal {L},x) + \delta $$,

where $G(K,\mathcal {L})$ denotes the maximal number of lattice vector any translate of *K* can contain, formally:

$$\begin{aligned} G(K, \mathcal {L}) = \max _{x \in \mathbb {R}^n}|(K+x)\cap \mathcal {L}|. \end{aligned}$$

By a covering argument (see [13, Lem. 2.3]), $G(dK,\mathcal {L})\le (2d+1)^n G(K,\mathcal {L})$. By the second condition, if $\mathcal {L'}$ is a $(K,\delta )$-sparsifier for $\mathcal {L}$, for every lattice point $v \in \mathcal {L}$, there is $v'\in \mathcal L'$ such that $\Vert v-v'\Vert _K \le \delta$. These two conditions ensure that the resulting lattice $\mathcal {L'}$ is thinned out according to the geometry of *K*: the first condition guarantees that *K* (or a dilate of *K*) cannot contain too many lattice vectors of $\mathcal {L'}$ (hence enumeration is not too costly), but, by the second condition, $\mathcal {L'}$ is rather close to $\mathcal {L}$ and thus serves as a good approximation.

We now come to the main observation:

### Lemma 5.2

Let *K* be an origin symmetric convex body with modulus of smoothness bounded by $\rho _K \le C\tau ^q$, $q \ge 1$, $\mathcal {L}$ a lattice and $t\in \mathbb {R}^n$ a target vector. Assume that $t + K$ does not contain any lattice vector $v \in \mathcal {L}$ in its interior. Let $\mathcal {L'}$ be a $(K,\epsilon ^{1/q})$ sparsifier for $\mathcal {L}$. Then

$$\begin{aligned} d_K(\mathcal {L'}, t) \le d_K(\mathcal {L},t) + 2C\epsilon . \end{aligned}$$

### Proof

Denote by $v \in \mathcal {L}$ a closest lattice vector to *t*, and set $R:=d_K(\mathcal {L},t)$. Clearly, $R=\left\| v-t\right\| _K\ge 1$. By the second condition of the sparsifier, there is a lattice vector $w \in \mathcal {L'}$ with $\left\| w-v\right\| _K\le \epsilon ^{1/q}$. Denoting by $y:=w-v \in \mathcal {L}$, the definition of the modulus of smoothness yields

$$\begin{aligned} \left\| \frac{w-t}{R}\right\| _K=\left\| \frac{v-t}{R}+\frac{y}{R}\right\| _K\le 2 + \frac{2C\epsilon }{R^q}-\left\| \frac{v-t}{R}-\frac{y}{R}\right\| _K \le 1 +\frac{2C\epsilon }{R^q}, \end{aligned}$$

where we used the fact that $v-y\in \mathcal {L}$, and hence, $\left\| (v-y)-t\right\| _K\ge R$. Multiplying the inequality by *R* and observing that $R,q\ge 1$ completes the proof of Lemma 5.2. $\square$

Next, we present the algorithmic application of the previous lemma to the $(1+\epsilon )$-approximate Closest Vector Problem under a symmetric norm. We adopt the same notation as in Sect. 4. We may assume that $t\in \mathbb {Z}^n$, $\mathcal {L}(A) \subseteq \mathbb {Z}^{n}$ and $\Vert t\Vert _{\infty },\Vert A\Vert _{\infty } \le 2^{(n^2 + n)b}$. We assume $n^{-3/2}B_2^n \subseteq K\subseteq ({1}/{2})B_2^n$. Thus, $d_K(\mathcal {L},t)\le 2n^{5/2}2^{(n^2 +n)b}$, and, if $t \notin \mathcal {L}(A)$, $t + K$ does not contain a lattice vector. We will need the following two algorithms.

### Theorem 5.3

($\texttt {Lattice-Enumerator}(K, t, \mathcal {L},\epsilon )$ [14]) Let $\mathcal {L}(A)$ be a lattice, *K* a convex body in $\mathbb {R}^n$ and $\epsilon >0$. There is a deterministic algorithm that outputs all *S* such that

$$\begin{aligned} (t + K) \cap \mathcal {L} \subseteq S \subseteq (t+K + \epsilon B_2^n)\cap \mathcal {L} \end{aligned}$$

in time $G(K,\mathcal {L})2^{O(n)}{{\,\mathrm{poly}\,}}(n,b)$ and $2^n{{\,\mathrm{poly}\,}}(n,b)$ space.

### Theorem 5.4

($\texttt {Lattice-Sparsifier}(\mathcal {L}(A), K, \delta )$ [13]) For $\delta > 0$, a basis $A'$ for a $(K,\delta )$-sparsifier for $\mathcal {L}(A)$ can be computed deterministically in $2^{O(n)}{{\,\mathrm{poly}\,}}(n,b)$ time and $2^n{{\,\mathrm{poly}\,}}(n,b)$ space.

We now combine these two theorems with Lemma 5.2.

### Theorem 5.5

There is an algorithm (described in the proof) that for an origin symmetric convex body *K* in $\mathbb {R}^n$, with modulus of smoothness bounded by $\rho _K \le C\tau ^q$ with some $C,q \ge 1$, solves $(1+\epsilon )$-$\text {CVP}_{K}$ for any lattice $\mathcal {L}$ and target vector $t\in \mathbb {R}^n$ in time $O(({C}/{\epsilon })^{n/q}){{\,\mathrm{poly}\,}}(n,b)$ and space $2^n {{\,\mathrm{poly}\,}}(n,b)$.

### Proof

We may assume $\epsilon \le 1$. If $t \in \mathcal {L}(A)$ (this can be checked in ${{\,\mathrm{poly}\,}}(n,b)$ time), return *t*. Else, set $\bar{\epsilon }={\epsilon }/({4C})$ and $d = 0$ and apply the following algorithm.

(i) Set $$K_d = 2^d K$$.
(ii) Apply $\texttt {Lattice-Sparsifier}(K_d,\mathcal {L},\bar{\epsilon }^{1/q}$). Denote the sparsified lattice by $\mathcal {L'}$.
(iii) Apply $\texttt {Lattice-Enumerator}((2+\epsilon )K_d, t,\mathcal {L'},\epsilon )$. If there is a lattice vector in $t + (2+\epsilon )K$, return the closest one to *t*, and stop. Else, set $d \leftarrow d+1$ and go to (i).

Let *k* be the largest positive integer such that $t + K_{k}$ does not contain a lattice vector. First, we claim that the algorithm will terminate at iteration $d\le k$. Indeed, since $t+2K_k=t+K_{k+1}$ contains a lattice vector of $\mathcal {L}$, by Lemma 5.2, $(2+\epsilon )K_k$ contains a lattice vector of $\mathcal {L'}$, and hence, the algorithm will terminate at $d=k$, or before.

To bound the error, we assume that the algorithm terminated at iteration *d*. By the previous paragraph, $t + K_{d}$ does not contain a lattice vector, and thus,

5 $$\begin{aligned} d_K(\mathcal {L}, t) \ge 2^{d}. \end{aligned}$$

Let *v* denote the lattice vector returned by $\texttt {Lattice-Enumerator}((2+\epsilon )K_d,t,\mathcal {L'},\epsilon )$. By Lemma 5.2, we only have an additive error of $2C\bar{\epsilon }={\epsilon }/{2}$ with respect to $\left\| \,{\cdot }\,\right\| _{K_d}$, that is,

$$\begin{aligned} d_{K_d}(\mathcal {L}, t)\le \left\| t-v\right\| _{K_d}+\frac{\epsilon }{2}, \end{aligned}$$

which, by (5) yields

$$\begin{aligned} d_{K}(\mathcal {L}, t)\le \left\| t-v\right\| _{K}+2^d\epsilon \le \left\| t-v\right\| _{K}+\epsilon d_{K}(\mathcal {L}, t), \end{aligned}$$

and hence $d_{K}(\mathcal {L}, t)\le \left\| t-v\right\| _{K}/{(1-\epsilon /2)}\le (1+\epsilon )\left\| t-v\right\| _{K}$. Thus, we found a $(1+\epsilon )$-approximate solution.

Next, we consider the time and space requirements. It is clear that step (ii) always takes time $2^{O(n)}{{\,\mathrm{poly}\,}}(n,b)$ and space $2^n{{\,\mathrm{poly}\,}}(n,b)$, independently of *d*. Note that $G((2+\epsilon )K, \mathcal {L'}) \le G(3K,\mathcal {L'})\le O({1}/{\bar{\epsilon }})^{n/q}$, and thus, step (iii) takes $O({C}/{\epsilon })^{n/q}{{\,\mathrm{poly}\,}}(n,b)$ time and $2^n {{\,\mathrm{poly}\,}}(n,b)$ space. Since $d_K(t,\mathcal {L})\le 2n^{5/2}2^{(n^2+ n)b}$, we need at most $\log _{2}(2n^{5/2}2^{(n^2 + n)b}) = {{\,\mathrm{poly}\,}}(n,b)$ iterations, resulting in time $O({C}/{\epsilon })^{n/q}{{\,\mathrm{poly}\,}}(n,b)$. This completes the proof of Theorem 5.5. $\square$

## Appendix: Proof of Some Lemmas

### Proof of Lemma 2.2

We cover *K* greedily by copies of $({\epsilon /2})K$ as follows. If after selecting $i-1$ homothetic copies of *K* there is a point $p_i \in K$ not yet covered, we take $Q_i=p_i+({\epsilon /2})K$. To see that after $N \le ({5/\epsilon })^n$ steps, all points of *K* are covered, we notice that the sets $({1}/{2})\odot Q_i$ are non-overlapping, and are contained in $(1+\epsilon /4)K \subseteq ({5}/{4})K$. Taking the volume of these sets, we obtain the desired bound. $\square$

### Proof of Lemma 2.3

Let $\{Q_i\}_{i=1}^N$ be a $(2,\epsilon )$-covering of *K*. For each $i\in [N]$, we will find a $(2,1)$-covering for $Q_i$ using at most $10^n$ centrally symmetric convex bodies. Thus, the union of these at most $10^nN$ symmetric sets will yield a $(2,\epsilon )$-covering of *K*. Fix $i\in [N]$ and set $\tilde{Q}_i=({1/2})((Q_i-c(Q_i))\cap (c(Q_i)-Q_i))$. In the same fashion as in the proof of Lemma 2.2, let $\{b_1,\ldots ,b_m\}$ be a maximal subset of $Q_i$ such that the interiors of the sets $b_1+({1}/{2})\tilde{Q}_i, \ldots , b_m+({1}/{2})\tilde{Q}_i$ are pairwise disjoint. Clearly, $\tilde{Q}_i+\{b_1,\ldots ,b_m\}$ is a covering of $Q_i$.

By a result of Milman and Pajor [26], if the centroid of a convex body *Q* in $\mathbb {R}^n$ is the origin, then

6 $$\begin{aligned} {{\,\mathrm{vol}\,}}{(Q\cap -Q) }\ge 2^{-n}{{\,\mathrm{vol}\,}}(Q). \end{aligned}$$

Thus, ${{\,\mathrm{vol}\,}}{(b_k + (1/2)\tilde{Q}_i)}\ge 8^{-n}{{\,\mathrm{vol}\,}}(Q)$, $k=1,\ldots ,m$. Since $b_k\in Q_i$ and $({1/2})\tilde{Q}_i\subseteq (1/4)(Q_i-c(Q_i))$, we have that $b_k + (1/2)\tilde{Q}_i\subseteq ({5}/{4})\odot Q_i$. Thus, $m\le 10^n$.

To see that $\tilde{Q}_i+\{b_1,\ldots ,b_m\}$ is $(2,1)$-covering of $Q_i$, note that $2\tilde{Q}_i\subseteq (Q_i-c(Q_i))$, and hence $b_k+2\tilde{Q}_i\subseteq Q_i+ (Q_i-c(Q_i)) = 2\odot Q_i$, as required. $\square$

### Proof of Lemma 2.4

The same argument as that used in the proof of Lemma 2.2 combined with (6) yields it. $\square$

### Proof of Proposition 2.5

We may assume that $\epsilon = (2^k-1)^{-1}$ for some positive integer *k*. For $i \in [k]$, the following union of translated intervals is a $(2,\epsilon )$-covering of $[-b,b]$:

$$\begin{aligned}{}[-b,b] \,\subseteq \! \bigcup _{\begin{array}{c} \delta \in \{\pm 1\}\\ j\in [k] \end{array}}\bigl (\delta (1- (2^j-1)\epsilon )b +[-2^{j-1}\epsilon b, 2^{j-1}\epsilon b]\bigr ). \end{aligned}$$

We may decompose analogously every line segment generating $\mathcal {Z}$ and combine them to give a $(2,\epsilon )$-covering for $\mathcal {Z}$:

$$\begin{aligned} \mathcal {Z}\, \subseteq \!\bigcup _{\begin{array}{c} \delta \in \{\pm 1\}^m\\ \alpha \in [k]^m \end{array}}\sum _{i=0}^{k}\,\bigl (\delta _i(1-(2^{\alpha _i}-1)\epsilon )b_i +[-2^{\alpha _i-1}\epsilon b_i, 2^{\alpha _i-1}\epsilon b_i]\bigr ). \end{aligned}$$

This is a $(2,\epsilon )$-covering for $\mathcal {Z}$ using $(2\log _2(1+1/\epsilon )+1)^m$ (translated) zonotopes. $\square$

### Proof of Proposition 2.6

We may assume that $\epsilon = ((4/3)^k-1)^{-1}$ for some positive integer *k*. For $\alpha \in [k]^m$ and $\delta \in \{\pm 1\}^m$, consider the following polytopes $\bar{Q}(\alpha ,\delta )$:

$$\begin{aligned}&\biggl \{x :\biggl (1-\biggl (\biggl (\frac{4}{3}\biggr )^{\!\alpha _i}-1\biggr )\epsilon \biggr )b_i\le \delta a_i^T x\le \biggl (1- \biggl (\biggl (\frac{4}{3}\biggr )^{\!\alpha _i-1}-1\biggr )\epsilon \biggr )b_i , i \in [m]\biggr \}. \end{aligned}$$

For each facet direction $|a_i^T x|\le b_i$, scaling each of the resulting (non-empty) $\bar{Q}$ around any point in its interior by a factor 4, it is straightforward to check that the resulting convex body is contained inside $\{x \in \mathbb {R}^n:|a_i^T x| \le (1+\epsilon )b_i\}$. It follows that each such non-empty polyhedron $\bar{Q}$ can be scaled by a factor 4 around any point in it and the resulting polytope is still contained inside $(1+\epsilon )P$ and it is clear that *P* is contained in the union of the $\bar{Q}(\alpha , \delta )$.

We could stop here and have a $(2,\epsilon )$-covering for *P*, but we are not guaranteed that the resulting cells are centrally symmetric. In order to ensure this, we will symmetrize the resulting $\bar{Q}(\alpha , \delta )$ as follows. Fix $x(\alpha ,\delta )\in \bar{Q}(\alpha ,\delta )$ and define

$$\begin{aligned} \bar{Q}_x(\alpha ,\delta ) = x(\alpha ,\delta ) +{{\,\mathrm{conv}\,}}{(\bar{Q}(\alpha ,\delta )-x(\alpha ,\delta ), x(\alpha ,\delta ) -\bar{Q}(\alpha ,\delta ))}. \end{aligned}$$

These are centrally symmetric polytopes with center of symmetry at $x(\alpha ,\delta )$. When $\bar{Q}$ is scaled by a factor 4, it is still contained in $(1+\epsilon )P$, thus we have $2\odot Q_x(\alpha ,\delta ) \subseteq (1+\epsilon )P$. Thus, the union of all $\{\bar{Q}_x(\alpha ,\delta )\}$ is a $(2,\epsilon )$-covering for *K* using at most $2^m(\log _{4/3}(1/\epsilon )+1)^m$ symmetric convex bodies. $\square$
